# A complete investigation of monocular and binocular functions in clinically treated amblyopia

**DOI:** 10.1038/s41598-017-11124-0

**Published:** 2017-09-06

**Authors:** Wuxiao Zhao, Wu-Li Jia, Ge Chen, Yan Luo, Borong Lin, Qing He, Zhong-Lin Lu, Min Li, Chang-Bing Huang

**Affiliations:** 1 Center for Optometry and Visual Science, Department of Optometry and Ophthalmology, The People’s Hospital of Guangxi Zhuang Autonomous Region, 6 Taoyuan Road, Qingxiu Dist, Nanning, 530021 P.R. China; 2 Department of Psychology, School of Education Science, Huaiyin Normal University, 111 Changjiang West Road, Huaian, 223300 P.R. China; 3 Key Laboratory of Behavioral Science, Institute of Psychology, CAS, 16 Lincui Road, Chaoyang Dist, Beijing, 100101 P.R. China; 4 Department of Psychology, University of the Chinese Academy of Sciences, 19A Yuquan Rd, Shijingshan District, Beijing, 100049 P.R. China; 5 Laboratory of Brain Processes (LOBES), Departments of Psychology, Center for Brain and Cognitive Sciences, Center for Cognitive and Behavioral Brain Imaging, The Ohio State University, Columbus, OH 43210 United States of America

## Abstract

The gold standard of a successful amblyopia treatment is full recovery of visual acuity (VA) in the amblyopic eye, but there has been no systematic study on both monocular and binocular visual functions. In this research, we aimed to quantify visual qualities with a variety of perceptual tasks in subjects with treated amblyopia. We found near stereoacuity and pAE dominance in binocular rivalry in “treated” amblyopia were largely comparable to those of normal subjects. CSF of the pAE remained deficient in high spatial frequencies. The binocular contrast summation ratio is significantly lower than normal standard. The interocular balance point is 34%, indicating that contrast in pAE is much less effective as the same contrast in pFE in binocular phase combination. Although VA, stereoacuity and binocular rivalry at low spatial frequency in treated amblyopes were normal or nearly normal, the pAE remained “lazy” in high frequency domain, binocular contrast summation, and interocular phase combination. Our results suggest that structured monocular and binocular training are necessary to fully recover deficient functions in amblyopia.

## Introduction

Successful treatment of amblyopia is important to improve the quality of life of those with the condition^1, 2^. The current gold standard of a successful amblyopia treatment is full recovery of visual acuity in the amblyopic eye, with the criterion ranging from 6/12 to 6/6 in different reports^3–6^. However, some evidence suggests that a number of visual functions remain deficient for patients with treated amblyopia, including contrast sensitivity (especially in high spatial frequencies)^5^, contrast visual acuity^7^, static aspects of accommodation^8^, fellow eye deficits^9^, and eye-hand coordination^10^. One area that remains to be systematically evaluated is binocular functions in treated amblyopia.

Human vision is binocular. Normal visual function requires comparable monocular functions as well as balanced interocular interactions^11–17^. Using different tasks and paradigms, previous studies have documented that amblyopia does not only impact monocular functions of the amblyopic and fellow eyes but also interocular interactions between the amblyopic and fellow eyes^9, 18–26^. It remains unclear if traditional treatments can recover both monocular and interocular functions in amblyopia. Most previous studies about treated amblyopia have focused on the quality of monocular functions in the amblyopic eye, e.g. visual acuity and contrast sensitivity. Since monocular functions in a given eye were usually tested when the untested eye was covered with an opaque patch, interocular interaction was minimized in the tests^19^. In other words, the seemingly normal monocular functions in treated amblyopia may not necessarily be normal in daily functions when both eyes are open and presented with similar luminance, and interact with each other.

In this study, we systematically evaluated a series of monocular and binocular functions in a group of subjects with treated amblyopia, aiming to provide a more complete picture of the functions of the pAE following successful amblyopia treatment and, in particular, test how the previously amblyopic eye behaves in binocular viewing conditions. Relative results will shed new light on the understanding and evaluation of amblyopia and amblyopia treatment.

## Methods

### Subjects

In the current study, three criteria were used to define treated amblyopia: (1) pAE achieves 0.18 logMAR (i.e. 6/9) or better visual acuity, (2) the visual acuity of the pAE and pFE differs by less than 2 lines, and (3) the improvements have been maintained for at least three months^27, 28^. Sixteen treated amblyopic patients (15.8 ± 2.3 yrs), most with histories of anisometropic amblyopia and six months to two years of patching treatment, participated in the study. Detailed characteristics of the subjects, including age, gender, optical correction, and their corrected visual acuity, stereoacuity, and interocular balance point in binocular phase combination (see below for details) are listed in Table 1. The study protocol was approved by the Institutional Review Board of the Institute of Psychology, Chinese Academy of Sciences, and the People's Hospital of Guangxi Zhuang Autonomous Region. This study adhered to the tenets of the Declaration of Helsinki and informed consent was obtained from the subjects after explanation.

**Table 1:** Characteristics of subjects.

No.	Sex	Age(yr)	AE Correction	AE Acuity (LogMAR)	FE Correction	FE Acuity (LogMAR)	Stereoacuity (log10(”))	Balance point*
1	F	24	+4.5DS: +0.5DC × 119	0.18	+3.75DS: +0.5DC × 59	−0.01	1.40	0.61
2	F	12	−2.25DS: −1.00DC × 170	−0.02	+2.00DS: +1.50DC × 90	−0.03	1.30	0.77
3	F	36	+1.50DS	−0.02	Plano	−0.03	1.30	0.34
4	M	10	+4.00DS: +0.50DC × 70	0	+1.25DS: +0.50DC × 75	−0.13	2.00	0.06
5	M	36	−15.00DS: −1.75DC × 10	0.09	−8.50DS: −3.50DC × 5	0.09	1.80	0.25
6	M	19	+3.00DS: + 1.50DC × 100	0.05	+3.00DS: +1.00DC × 80	−0.02	2.00	0.28
7	M	20	+0.25DS: +1.25DC × 90	0.07	+0.5DS: +0.75DC × 90	0.07	2.20	0.39
8	F	10	+1.00DS	0.11	+1.25DS	0.07	1.40	0.01
9	M	7	+3.50DS: +0.50DC × 90	−0.03	+1.50DS	−0.02	1.30	0.19
10	M	15	−1.50DS: −0.50DC × 160	−0.02	−1.50DS: −1.00DC × 165	−0.13	1.80	0.47
11	F	9	+1.00DC × 55	−0.03	+1.00DS: −1.75DC × 15	−0.02	1.30	0.47
12	M	10	+2.5DC × 85	−0.03	+2.25DC × 95	−0.03	2.00	0.62
13	M	14	−5.00DC × 170	0.03	+1.50DS: −5.00DC × 175	0.03	1.30	0.62
14	F	10	−5.50DS: −1.75DC × 170	0.07	−5.50DS: −2.00DC × 10	0.03	1.60	0.57
15	F	10	+2.50DS: +0.50DC × 90	0.07	+0.50DC × 80	0.07	1.30	0.48
16	F	11	+3.0DS: +1.0 DC × 35	0.03	−05DS: −0.5DC × 175	−0.07	1.40	0.06

*Balance point in binocular phase combination.

### Apparatus

Except some standard tests, stimuli were generated using a computer running Matlab 8.0 based on Psychtoolbox extensions 3.0^29, 30^ and presented on a gamma-corrected Sony G220 color monitor (21 inch, P22 phosphor; Sony, Tokyo, Japan). The spatial resolution of the monitor was 1600 × 1200 pixels, the vertical refresh rate was 85 Hz, and the mean luminance was 28.3 cd/m^2^. We used a special circuit to produce 14-bit gray-level resolution^31^. Subjects viewed the displays in fovea in a dimly light room at a distance of 1.38 meters. A chin rest was used to stabilize subject’s head during the experiment.

### Visual acuity

Monocular visual acuity (VA) was measured for both eyes using a Chinese Tumbling E chart^32^ with the untested eye covered by an opaque patch and specified as logMAR^33^.

### Contrast sensitivity function (CSF)

**Figure 1:**
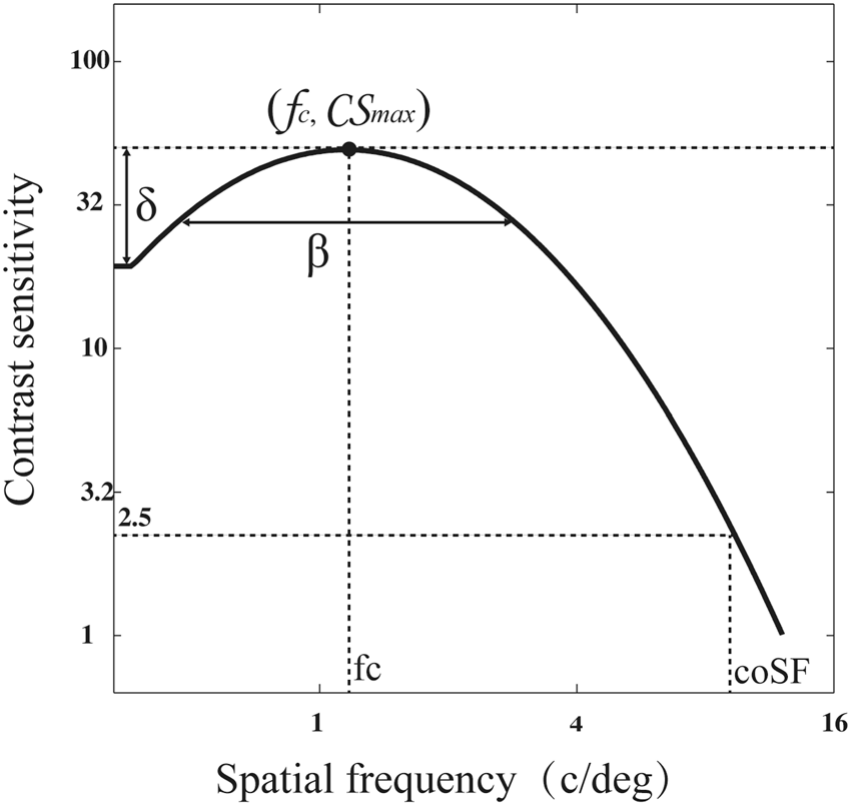
CSF Parameterization. The spatial contrast sensitivity function, which describes the reciprocal of contrast threshold as a function of spatial frequency, can be described by four parameters^34^: (1) peak gain, *CS*_*max*_, (2) peak frequency, *f*_*c*_, (3) bandwidth (full width at half-maximum), β, and (4) truncated fall-off on the low-frequency side, δ. The qCSF method rapidly estimates the CSF by directly estimating these four parameters.

Monocular and binocular contrast sensitivity functions, a more comprehensive evaluation of spatial vision, was estimated using the quick CSF method (Fig. 1)^34^. In monocular testing, the untested eye was covered by an opaque patch. The stimuli were 2.5° × 2.5° vertical sine-wave gratings. A half-Gaussian ramp (σ = 0.25°) was added to each edge of the gratings to minimize edge effects. The stimulus space consisted of gratings with contrasts ranging from 0.1% to 99% in steps of 1.5 dBs and spatial frequencies from 0.5 to 16 cpd in steps of 3 dBs. Each CSF was obtained with 100 quick CSF trials in a two-interval forced choice (2IFC) paradigm. Each trial consisted of an initial 294-ms fixation in the center of the display and two 153-ms stimulus intervals separated by an inter-stimulus interval (ISI) of 588 ms. A brief tone signaled the onset of each interval. The grating was only presented in one of the two intervals. Subjects were asked to indicate the interval that contained the grating using a computer key press. No feedback was provided.

Two indices were derived from the quick CSF test, the area under the log contrast sensitivity function (AULCSF) and cutoff spatial frequency (coSF). The AULCSF was calculated by integrating CSF in the range from 0.5 to 16 c/deg, and the coSF was defined as the spatial frequency at which the contrast sensitivity was 2.5 (i.e., contrast threshold of 0.4).

### Stereoacuity

Stereoacuity was assessed with the Fly Stereo Acuity Test that consisted of 10 circles ranging from 400 to 20 arcsecs (Fly Stereo Acuity Test; Vision Assessment Corporation, IL). We administered the test according to the manufacture’s guidelines. Subjects looked directly at the test material at a viewing distance of 40 cm and reported which circle was out of the plane of the other three (zero plane). We started the test with the easiest condition (fly of 4800”) and gradually increased its difficulty. Test materials were also rotated 90 degrees to ensure that the judgments were based on stereo perception. The light level during the test was kept approximately the same across subjects.

### Worth 4-dot test

The Worth 4-Dot test was used to assess eye dominance. Under moderate room illumination, subjects wore red/green anaglyph glasses with the red filter over the right eye and the green filter over the left eye and were instructed to fixate on the Worth 4-Dot stimulus (diameter = 8.5 mm; 2 green, 1 yellow, and 1 red with equal luminance, and with the yellow dot at the bottom) while it was held slightly below the participant’s line of sight at a distance of 33 cm. Eye dominance was determined based on the perceived color of the yellow dot. Subjects were asked to first report how many dots they perceive and then make a three-alternative, forced-choice decision about whether the bottom dot appeared yellow or red and green (i.e. no dominance), red (right eye dominant), or green (left eye dominant).

### Binocular phase combination

**Figure 2:**
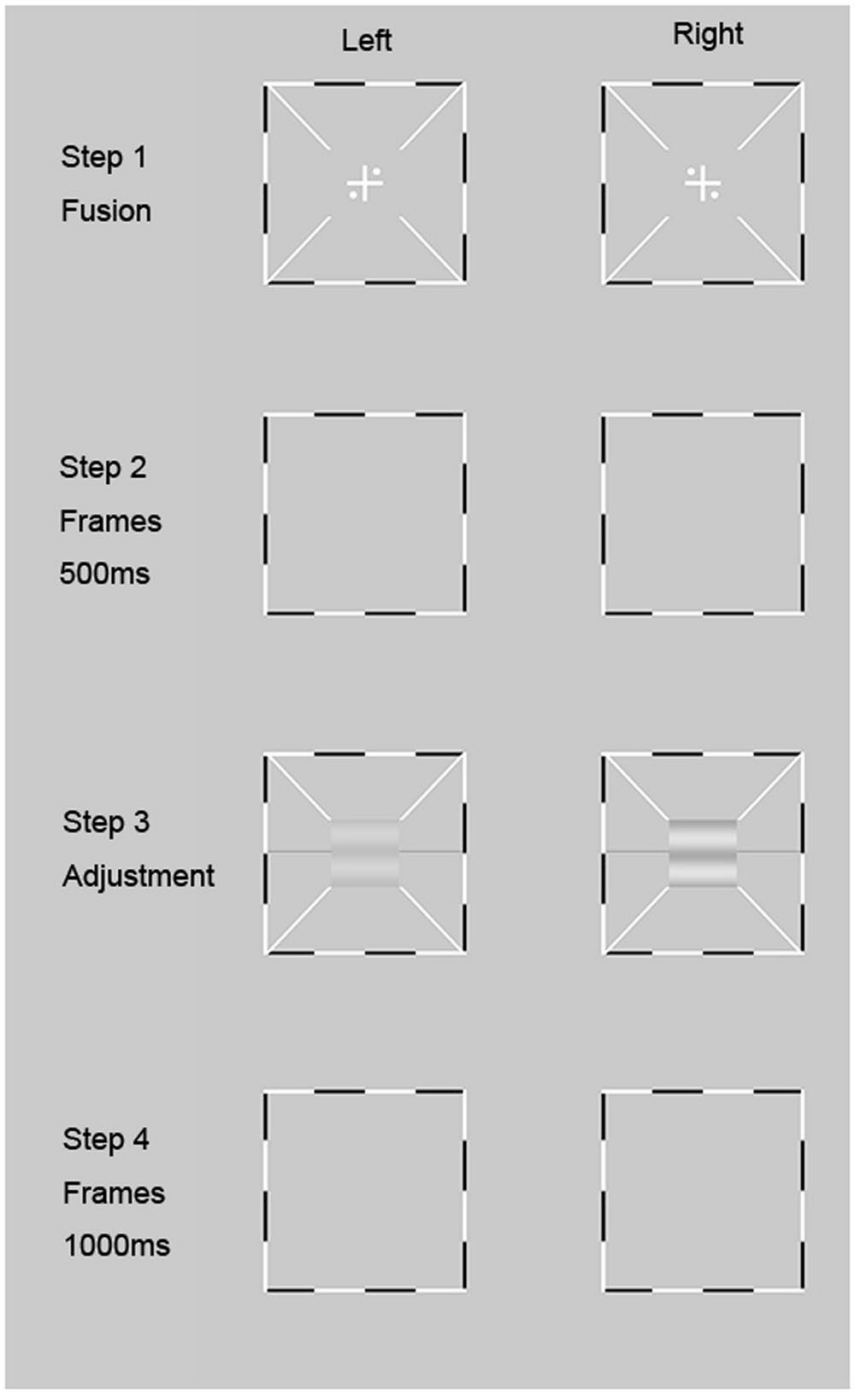
Illustration of the binocular phase combination test^21^. Step 1: Two frames were dichoptically displayed to the left and right eyes and subjects adjusted the stereoscope to fuse the two into a cross with four balanced dots; Step 2: pre-stimulus interval of 500 ms; Step 3: two sine-wave gratings were displayed and subjects were asked to report the center of the dark stripe of the cyclopean grating by moving the horizontal line; Step 4: inter-trial interval of 1000 ms.

The Worth 4-dot test provides a qualitative measure of sensory eye dominance. To quantify interocular interaction between the two eyes, we adopted a suprathreshold binocular phase combination paradigm (Fig. 2)^21, 26, 35^. The test stimuli consisted of two horizontal, 45 deg out-of-phase sine-wave gratings of 1.0 c/deg, each subtending 2 × 2 deg^2^ at a viewing distance of 1.38 m. The contrast of the stimuli presented to the pAE was fixed at C_A_ = 50%; The contrast of the stimuli in the pFE varied, with the interocular contrast ratio C_F_/C_A_ ∈ [0, 0.1, 0.2, 0.4, 0.8, 1]. Two phase-configurations were tested. In one configuration, the phase of the grating in the pAE was 22.5 deg and that in the pFE was −22.5 deg. In the other configuration, the phase of the grating in the pAE was −22.5 deg and that in the pFE was  22.5 deg. Results from the two configurations were combined to eliminate bias in phase judgments^26^. A stereoscope was used to direct the images of the two gratings to the appropriate eyes. Subjects were asked to report the perceived phase of the binocularly-combined cyclopean sine-wave grating (Fig. 2). To assist good binocular fusion, the grating in each eye was placed in the center of a larger (6 × 6 deg^2^), high-contrast frame with clearly marked white diagonals (Fig. 2). 96 trials were tested in 12 conditions (6 interocular contrast ratios × 2 phase configurations), with 8 trials in each condition.

Each trial started with the presentation of the binocular fixation crosses, the high contrast frames, and the monocular fixation dots. Subjects adjusted the stereoscope to fuse the frames, the fixation crosses, and the monocular fixation dots. They then pressed the “space” bar on the computer keyboard to initiate the test after achieving stable vergence. This was followed by a 500-ms presentation of the frames, and then signal sine-wave gratings in the two eyes. To signal the onset of the sine wave gratings and help observers to fuse, the gratings were shown simultaneously with the diagonals of the frames and the reference lines in both eyes. Subjects were asked to adjust the location of a horizontal reference line to indicate the perceived phase of the cyclopean sine-wave grating, defined as the location of the center of the dark stripe of the grating, and press the “Enter” key after they finished the task. Each trial was followed by a 1-sec blank display. A typical trial lasted about 5 seconds.

In the end, we obtained a “PvC” curve, i.e. the perceived phase of the cyclopean grating versus contrast ratio of the gratings in the two eyes. Since gratings in the two eyes were opposite in phase (±22.5), a percieved phase of 0 deg of the cyclopean grating signaled equal contribution from the two eyes during binocular combination. We thus defined the interocular contrast ratio at which the perceived phase is zero as the interocular balance point. The lower the interocular balance point was, the more severe the imbalance between the two eyes. A ratio of 0 indicates full dominance of the fellow eye and 1 indicates perfect balance between the two eyes.

### Binocular rivalry

**Figure 3:**
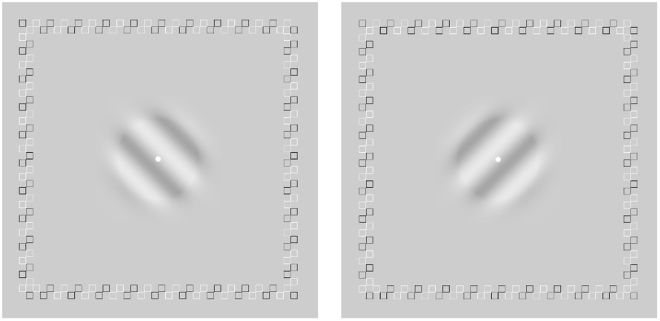
Illustration of the stimulus used in the binocular rivalry test.

Two orthogonal gratings (±45 degrees) of 40% contrast, each subtending 3 × 3 deg^2^, were presented to the two eyes dichoptically with a stereoscope at a viewing distance of 1.38 meters (Fig. 3). With a 0.25-deg half-Gaussian ramp on their edges, the part of the gratings with maximum contrast subtended 2.5 × 2.5 deg^2^. To help fuse the dichoptic displays, a high-contrast frame made of small open squares was used. The center fixation dot (lum = 56.6 cd/m^2^) was displayed throughout the experiment. Two frequency conditions, 1 c/deg and 8 c/deg, were tested with 4 trials each. In each trial, subjects viewed the dichoptic images for 120 seconds and reported their dominant percept by pressing one of two keys. The detailed time stamps of all dominant phases were recorded. The total dominance ratio between the pAE and pFE was computed from their respective dominance durations across all trials and used to index sensory dominance between the two eyes. A dominance ratio of 0 means complete dominance of the fellow eye and a ratio of 1 means equal dominance between the two eyes. The number of dominance switch between the two eyes was also tabulated. Trials for different frequency conditions were intermixed.

### Binocular summation ratio

Following tradition, we termed the ratio in the area under log CSF (AULCSF) between the binocular and the monocular pFE testing conditions as the binocular summation ratio^13, 36^. Previous studies showed that, with similar monocular contrast in the two eyes, the binocular contrast summation ratio was around 1.4 in normal subjects^13^ and approximately 1 (no significant binocular summation) in subjects with amblyopia^37–39^.

## Results

### Visual acuity

Although visual acuity in the pAE and pFE still differed significantly (t(15) = 2.78, p = 0.014), their difference was very small (0.035 vs −0.006 logMAR), indicating that the pAE and pFE were comparable in terms of visual acuity^4^.

### Binocular summation ratio

The average binocular summation ratio of the treated amblyopes was significantly lower than that of the normals reported in the literature (1.07 ± 0.03 vs 1.4, p < 0.001)^13^.

### Stereoacuity

The average stereoacuity of the treated amblyopes was 1.56 ± 0.08 log arc seconds (51.3 ± 10.7 arc seconds), comparable to the 1.6 log arc seconds (40 arc seconds) standard for normal vision (t(15) = 0.18, p = 0.86)^40^.

### Worth 4-dot test

**Figure 4:**
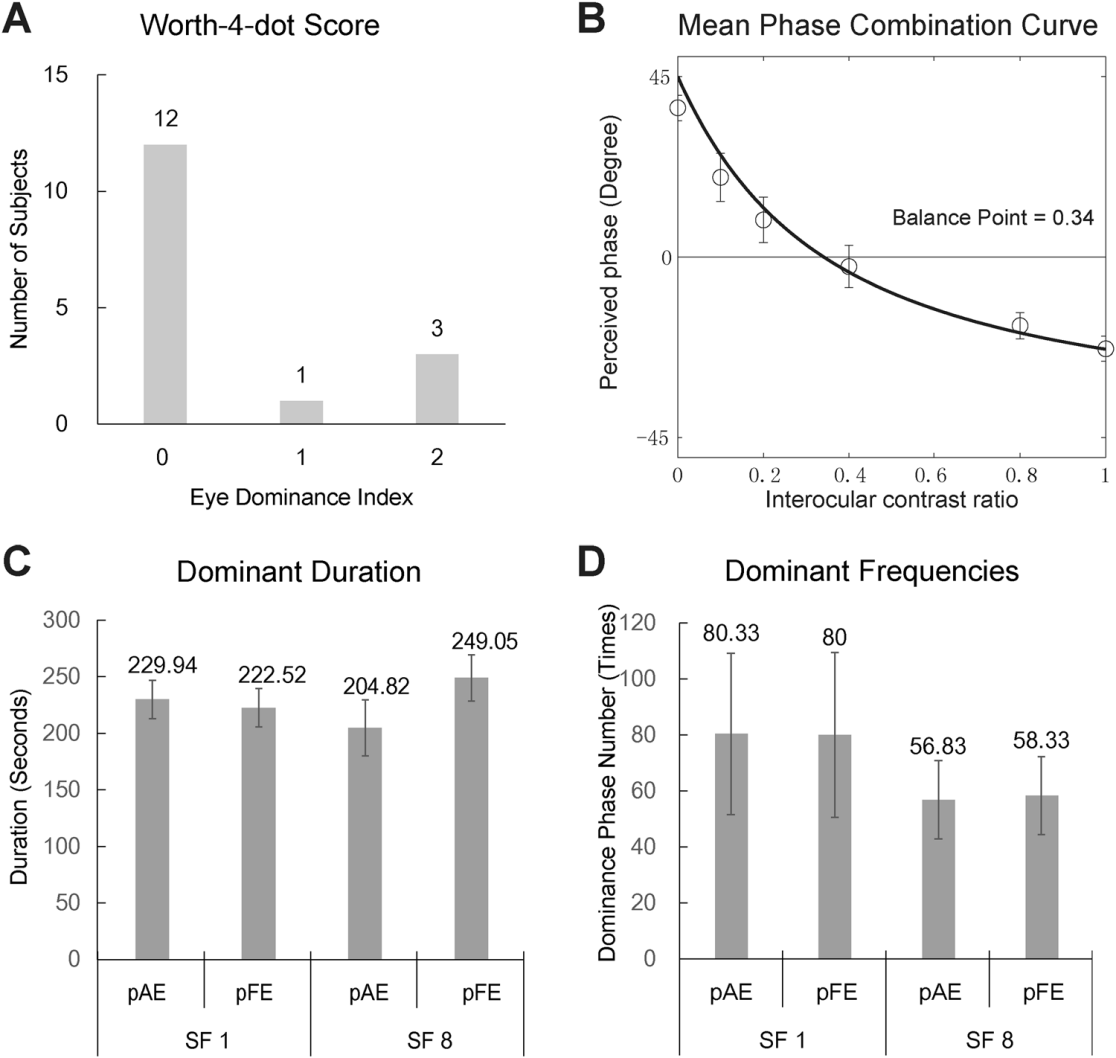
Results from the Worth-4-dot (**A**), binocular phase combination (**B**), and binocular rivalry tests (**C**,**D**).

Three subjects’ pFE showed full dominance and one subject’s pFE showed dominance once in two tests. The other twelve subjects showed perfect balance between the two eyes (Fig. 4A).

### Interocular balance point in binocular phase combination

The average interocular balance point was 0.34 ± 0.06 (Fig. 4B). The result suggested that, on average, the contrast of an image in the pAE was only equivalent to 34% of that in the pFE in binocular phase combination. There is no correlation between age and the balance point in binocular phase combination (R = −0.01, *p* > 0.10).

### Dominance ratio in rivalry

Six patients (21.2 ± 5.3 years) participated in the binocular rivalry test. In both test conditions, the pAE and pFE were essentially equivalent in terms of both the dominance duration and frequency: At 1 c/d, the pAE dominated 51% of the time (229.9 out of 452.5 seconds, Fig. 4C, p = 0.816) and 50% of the dominance episodes (80 out of 160 times, Fig. 4D, p = 0.741); at 8 c/d, it dominated 45% of the time (204.8 out of 453.9 seconds, Fig. 4C, p = 0.326) and 49% of the episodes (57 out of 115 times, Fig. 4D, p = 0.456).

### Pair-wise correlations between interocular and binocular visual functions

We computed pair-wise correlations between interocular difference in visual acuity/AULCSF/cutoff spatial frequency, binocular summation ratio, stereoacuity, Worth-4-dot and interocular balance point (Table 2). Significant correlation was only found between interocular difference in AULCSF and interocular difference in cutoff spatial frequency (Person’s R = 0.788, p = 0.02 after Bonferroni correction); none of the other correlations was significant (all p > 0.1 after Bonferroni correction).

**Table 2:** Correlation between monocular and binocular visual functions.

	Interocular Differences of			Binocular Summation Ratio	Stereoacuity	Worth-4-dot	Interocular Balance Point
	Visual Acuity	AULCSF	Cutoff Spatial Frequency				
Interocular Differences of Visual Acuity							
Interocular Differences of AULCSF	0.186(0.491)						
Interocular Differences of Cutoff Spatial Frequency	0.208(0.440)	**0.788(0.000)** ^**^					
Binocular Summation Ratio	0.149(0.581)	0.235(0.382)	0.216(0.421)				
Stereoacuity	0.055(0.840)	0.060(0.825)	0.405(0.120)	0.015(0.955)			
Worth-4-dot	0.448(0.082)	0.472(0.065)	0.287(0.282)	0.319(0.228)	0.028(0.917)		
Interocular Balance Point	0.178(0.509)	0.320(0.226)	0.287(0.281)	0.270(0.312)	0.114(0.675)	**0.500(0.049)** ^*^	

Pearson correlations were performed and expressed as “*R*(*p*)”. ^*^Correlation is significant; ^**^highly significant.

## Discussion

Visual acuity is widely used in daily diagnosis of amblyopia and most valued by clinicians^41, 42^. The current study demonstrates that although long-term occlusion can recover some of the visual functions in patients with amblyopia, including visual acuity, near stereo vision, and binocular rivalry at low spatial frequency, many other binocular visual functions remain deficient, including binocular rivalry at high spatial frequencies, interocular summation, and interocular phase combination. The results suggest that the occlusion treatment of amblyopia is not sufficient to recover all the visual functions in amblyopia; additional treatment is necessary.

Some studies have found that intensive perceptual learning can significantly improve the contrast sensitivity of the amblyopic eye at high spatial frequencies^20, 43–45^, its contribution in binocular phase combination^46^, and stereo vision^47–49^. In ongoing research, we are evaluating the efficacy of these methods in clinically “treated” amblyopia.

Our results also highlight some degree of independence among different visual functions in treated amblyopia. First, normal acuity does not necessarily mean normal contrast sensitivity. As we pointed out in Huang *et al*.^5^, identification of the orientation of letter E in the tumbling E test may be based on information from a broad range of spatial frequencies^5^. In this study, contrast sensitivity in the pAE and pFE was comparable at low to medium spatial frequencies, which may lead to comparable visual acuity in the two eyes, although contrast sensitivity in high spatial frequencies differed in the two eyes. Second, binocular rivalry, binocular phase combination, and binocular contrast summation may reflect (at least partially) different neural underpinnings since training can largely recover pAE’s performance in binocular rivalry in low spatial frequencies but not in binocular summer and binocular phase combination. Rivalry happens when the two eyes receive incompatible stimuli; binocular phase combination involves two stimuli of slight difference; binocular contrast summation involves two identical stimuli.

Amblyopia can result from attenuation of the signal in the amblyopic eye (monocular mechanism), abnormal interaction between the fellow and the amblyopic eyes (i.e. stronger suppression; binocular mechanism), or a combination of both^35^. The amblyopic vision of different patients may involve different mechanism(s)^35, 50^. A successful treatment of amblyopia should rescue all deficient mechanim(s), if present. Although it’s tempting to infer that the monocular mechanism was rescued in clinically treated amblyopia since visual acuity and contrast sensitivity at low to medium frequencies in the amblyopic eye were comparable to those in the fellow eye and some of the binocular functions were also normalized (e.g. rivalry at low frequency), we decide not to make the conclusion at this point and leave the answer to an ongoing project that measures both binocular contrast and phase combination in treated amblyopia, which will directly separate monocular and binocular contributions in amblyopic deficits^35^.

In summary, our results suggest systematic evaluations of visual functions are important in monitoring amblyopia treatment, and structured monocular and binocular training are necessary to fully restore deficient visual functions in amblyopia.
